# Destined to die in hospital? Systematic review and meta-analysis of place of death in haematological malignancy

**DOI:** 10.1186/1472-684X-9-9

**Published:** 2010-06-01

**Authors:** Debra A Howell, Eve Roman, Helen Cox, Alexandra G Smith, Russell Patmore, Anne C Garry, Martin R Howard

**Affiliations:** 1 Epidemiology and Genetics Unit, Department of Health Sciences, Seebohm Rowntree Building, University of York, YO10 5DD, UK; 2 Department of Haematology, Queens Centre for Oncology and Haematology, Castle Hill Hospital, Castle Road, Cottingham, East Yorkshire, HU16 5JQ, UK; 3 Palliative Medicine, North Yorkshire and York PCT, York, YO31 8HE, UK; 4 Department of Haematology, York Hospital, Wigginton Road, York, YO31 8HE, UK

## Abstract

**Background:**

Haematological malignancies are a common, heterogeneous and complex group of diseases that are often associated with poor outcomes despite intensive treatment. Research surrounding end-of-life issues, and particularly place of death, is therefore of paramount importance, yet place of death has not been formally reviewed in these patients.

**Methods:**

A systematic literature review and meta-analysis was undertaken using PubMed to identify all studies published between 1966 and 2010. Studies examining place of death in adult haematology patients, using routinely compiled morbidity and mortality data and providing results specific to this disease were included. 21 studies were identified with descriptive and/or risk-estimate data; 17 were included in a meta-analysis.

**Results:**

Compared to other cancer deaths, haematology patients were more than twice as likely to die in hospital (Odds Ratio 2.25 [95% Confidence Intervals, 2.07-2.44]).

**Conclusion:**

Home is generally considered the preferred place of death but haematology patients usually die in hospital. This has implications for patients who may not be dying where they wish, and also health commissioners who may be funding costly end-of-life care in inappropriate acute hospital settings. More research is needed about preferred place of care for haematology patients, reasons for hospital deaths, and how these can be avoided if home death is preferred.

## Background

Haematological malignancies are common, being the fourth most frequently diagnosed cancer in both males and females in economically developed regions of the world [1-3]. Although traditionally regarded as lymphoma, leukaemia and myeloma, haematological malignancies are, in fact, exceptionally heterogeneous, with the World Health Organisation classification system recognising over 60 different clinical and pathological disease subtypes [4]. This complexity is further reflected in the widely varying clinical features, treatment pathways and outcomes associated with these diseases [5]. Despite recent advances in treatment, survival for some disease subtypes may be poor, and in this context it is clear that good end-of-life care is an issue that is of equal significance as achieving cure [6]. Consequently, research surrounding end-of-life issues and particularly place of death is important, yet despite this, there is a distinct lack of formal research in this area.

It is generally believed that the majority of people, including haematology patients, would prefer to be cared for and die at home [7-9]. In the United Kingdom (UK), the National End of Life Care Programme [10] was launched in 2004, with one of its aims being to ensure individuals have more choice as to where they live and die. Publication of the End of Life Care Strategy [11] brought together initiatives from this programme including Advance Care Planning, Preferred Priorities for Care, the Gold Standards Framework and Liverpool Care Pathway for the Dying Patient, to ensure choice was available to patients and so enable them die in their preferred place.

A number of individual studies have examined place of death in patients with haematological malignancies [12-31,48]. Although these report patients dying in hospital more frequently than those with solid tumours, this has never been formally reviewed. This review systematically examines all studies of place of death in haematology patients and includes a meta-analysis of risk estimates. It also explores factors reported in the wider literature that are considered to lead to hospital deaths in these patients. Finally, further research is suggested, which would improve understanding of end-of-life care and place of death and could also be used to drive change in this complex area.

## Methods

PubMed was searched in January 2009 (with a final search in March 2010) for all studies, in any language, published between 1966 and 2009 and containing the terms 'haematology', 'lymphoma', 'leukaemia', 'myeloma' or 'cancer', combined with 'place of death', 'place of care' or 'end-of-life' in the title, abstract or keywords. PubMed titles were also searched for specific phrases including: 'place of death', 'death in hospital', 'nursing home death', 'hospice death', 'death at home', 'place end of life', 'dying in hospital', 'dying home' and 'dying hospice'.

All identified abstracts were reviewed by two researchers and a scoring system used to select papers meeting specific pre-determined criteria. One point was scored for each of the following: examining place of death; including patients with haematological malignancies; including adults; obtaining information on place of death from routinely compiled morbidity and mortality data; and providing results specific to the haematology patients. References of identified studies were also searched and additional papers included as appropriate. Studies of children ≤18 years were excluded due to the different issues associated with place of death in this age group in terms of disease types, clinical care, associated infrastructure (for example hospice provision) and family support.

**Figure 1:**
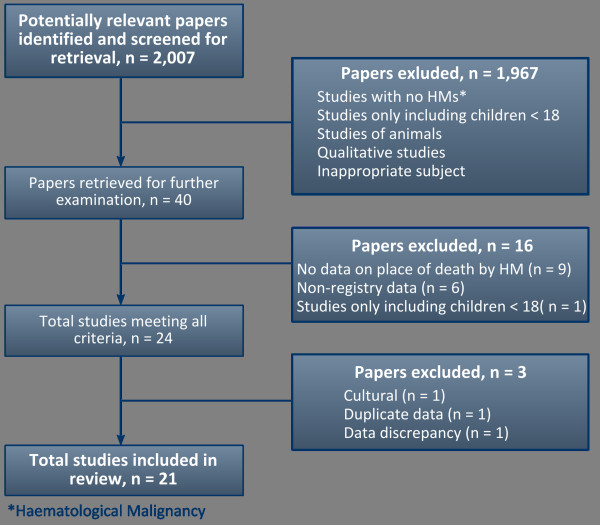
**Study Selection Process**.

The search identified 2,007 published reports (Figure 1). During the screening process, studies scoring 3 or less were excluded from the abstract alone (n = 1,927). Studies scoring 4 or 5, or with unclear scores (for example if the cancer sites included were not specified in the abstract) were retrieved for scrutiny (n = 80). Twenty-four publications meeting all the criteria were selected for final screening and a further 3 were then excluded. This was either because the data appeared elsewhere [32], or the study was in an area where important cultural differences influenced place of death to an extent not found in other settings [33], or data discrepancies were identified meaning that the original published material could not be used [34]. The remaining 21 studies are included in this review; and 17 presented data that could be included in a meta-analysis, one of which gave data for 5 individual countries. This equates to a total of over 30,150 haematology patients - the number of patients was not given in four studies, one of which was included in the meta-analysis [22].

**Figure 2:**
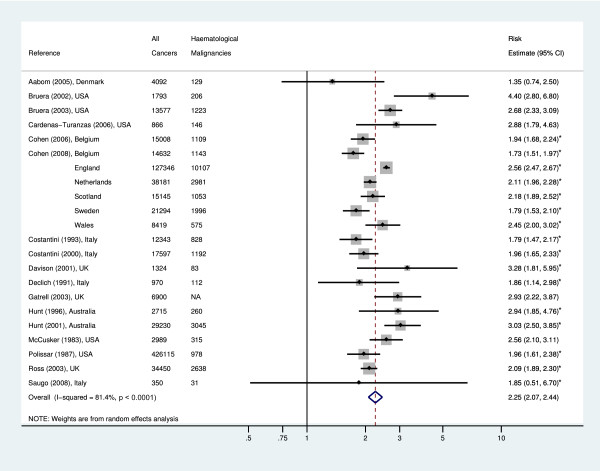
**Estimated risk of hospital death - haematological malignancy compared to non-haematolgical malignancy**. NA = Not Available. * Denotes risk-estimate calculated from data reported in study.

Descriptive data, in terms of the percentage of hospital deaths were extracted for all cancers and for haematological malignancies where given. Risk-estimates for hospital death, with 95% confidence intervals, were also extracted or calculated if data were amenable. Descriptive data were summarised and a meta-analysis of risk-estimates created using Stata 10.0 Statistical Software, and presented in a meta-plot (Figure 2).

## Results

In comparing haematological malignancies to other cancer deaths, it is evident that haematology patients are more likely to die in hospital (Table 1). As shown in Figure 2 haematology patients are, in fact, more than twice as likely to die in hospital compared to those with other cancers (Odds Ratio 2.25 [95% Confidence intervals, 2.07-2.44] p < 0.0001). Studies examining death in other areas, such as hospices, also found that those with haematological disease were less likely to die in these places compared to those with other cancers (Table 1) [20,26,30].

**Table 1:** Summary of reported findings and proportion of hospital deaths for all cancers and haematological malignancies.

Study	Country	Dates of deaths	Total patients (% hospital deaths)		Main findings
				
			All cancers	Haematological malignancy	
**Aabom, 2005 **[12]*	Denmark	01/96-12/98	4,092 (70)	129 (84)	Compared to all other cancer deaths, patients dying from 'haematological' cancers (coding not specified) were more likely to die in institutions (hospital/nursing home).

**Bruera, 2002 **[13]*	USA	09/97-08/98	1,466 (57)	206 (84)	Compared to all other cancer deaths, patients dying from 'hematologic' (coding not specified) were more likely to die in hospital.

**Bruera, 2003 **[14]*	USA	09/96-08/98	13,577 (51)	1,223 (NA)	Compared to all other cancer deaths, patients dying from 'haematological' cancers (coding not specified) were more likely to die in hospital.

**Cardenas-Turanzas, 2006 **[15]*	USA	1999-2000	866 (58)	146 (79)	Compared to all other cancer deaths, patients dying from 'leukaemia and lymphoma' were more likely to die in hospital than elsewhere (home/nursing home/hospice).

**Cohen, 2006 **[16]*	Belgium	2001	15,0008 (54)	1,109 (75)	Compared to all other cancer deaths, patients dying from 'hematologic' cancers were more likely to die in hospital than home/care home/elsewhere.

^**1**^**Cohen, 2008 **[17] *	Belgium Netherlands Sweden Scotland England Wales	2003 2003 2002 2003 2003 2003	14,632 (59) 38,181 (31) 21,294 (85) 15,145 (57) 127,346 (49) 8,419 (60)	1,143 (71) 2,981 (47) 1,996 (91) 1,053 (74) 10,107 (70) 575 (78)	Compared to all other cancer deaths, patients dying from 'hematologic malignancies' were more likely to die in hospital/care home than outside hospital. This was consistent across all countries included.

**Costantini, 1993 **[18]*	Italy	01/86-12/90	12,315 (69)	828 (79)	Compared to all other cancer deaths, patients dying from 'leukaemia-lymphoma' were more likely to die in an institution (hospital/elderly care home).

**Costantini, 2000 **[19]*	Italy	1991	17,597 (48)	1,192 (64)	Compared to all other specified cancer deaths, patients dying from cancers of the 'haemopoietic system' were more likely to die in a hospital/elderly care home.

**Davison, 2001 **[20]*	UK	July-Dec 1977/87/97	1,324 (47)	83 (75)	Compared to all other cancer deaths, patients dying from cancer of the 'lymphatic' and 'haemopoietic tissue' were more likely to die in hospital and less likely to die in a hospice.

**Declich, 1991 **[21]*	Italy	01/85-12/88	970 (18)	112 (27)	Compared to all other cancer deaths, patients dying from 'lymphoma' and 'haematopoietic neoplasms' were more likely to die in hospital (with the exception of colon cancer).

**Decker, 2006 **[48]	UK/USA	1995-1998	UK 59,604 (56) USA 51,668 (74)	NA	Compared to all other cancer deaths, patients dying from cancer of the 'lymphatic' and 'haematopoietic' tissue aged ≥40 years were less likely to die at home (12% UK; 14% USA).

**Gatrell, 2003 **[22]*	UK	1993-2000	6,900 (35)	NA	Compared to all other cancer deaths, patients dying from 'lymphatic system' cancer were more likely to die in hospital.

**Higginson, 1998 **[23]	UK	1985-1994	1,344,187 (66)	NA	Compared to all other cancer deaths, patients dying from cancers of the 'lymphatic' or 'haematological system' were less likely to die at home.

**Hunt, 1996 **[24]*	Australia	1990	2,800 (NA)	260 (NA)	Compared to all other cancer deaths, patients dying from 'haematological' cancers (coding not specified) were more likely to die in a Metropolitan Public Hospital.

**Hunt, 2001 **[25]*	Australia	1990-1999	29,230 (55)	3,045 (NA)	Compared to all other cancer deaths, patients dying from 'lymphoma', 'multiple myeloma' and 'leukaemias' (coding not specified) were more likely to die in a Metropolitan Public Hospital.

**Lock, 2005 **[26]	UK	1995-1999	315,462 (50)	NA (66)	Compared to all other cancer deaths, patients dying from 'lymphatic' and 'haematopoietic' cancers aged ≥75 years, were more likely to die in hospital and less likely to die in a hospice.

**McCusker, 1983 **[27]*	USA	1976-1978	2,989 (70)	315 (82)	Compared to all other cancer deaths, patients dying from 'leukaemia and lymphoma' (coding not specified) were more likely to die in an acute care hospital.

**Polissar, 1987 **[28]*	USA	1968-1981	22,456 (61)	978 (73)	Compared to other common cancers (9 selected cancer sites) patients with 'non-Hodgkin lymphoma' were the most likely to die in hospital.

**Roder, 1987 **[29]	Australia	1981 & 1985	1,582 (37)	NA (57)	Compared to other common cancers (8 selected sites), patients with 'haematological malignancies' (leukaemia, lymphoma and myeloma) were more likely to die in a Metropolitan Public Hospital.

**Ross, 2007 **[30]*	UK	1995-2000	31,812 (41)	2,638 (62)	Compared to all other cancer deaths, patients dying from haematological malignancy (all diagnoses combined) were more likely to die in hospital and less likely to die in a hospice.

**Saugo, 2008 **[31]*	Italy	2004	350 (75)	31 (87)	Compared to all other cancer deaths, patients dying from 'haematological' cancers ≥50 years were more likely to die in hospital.

^1 ^Data on place of death only given for hospital and care home combined with the exception of Sweden, where care home is not a registered category.

NA = not available; *Included in meta-analysis.

Variation was seen in the type of haematological malignancy included in individual studies, with some including several diagnostic categories and others focusing on one particular classification. However, results were again found to be largely consistent across sub-groups. One UK study with a detailed sub-group analysis reported more hospital deaths among all types of haematological malignancy (62%) compared to other malignancies (41%) but with some variation between subgroups [30]. For example, greater proportions of hospital deaths were seen in patients with acute myeloid leukaemia (67%) and chronic lymphocyte leukaemia (67%). Interestingly, the proportion of patients with acute lymphoblastic leukaemia dying at home (31%) was similar to that seen for other cancers (30%), as was the proportion with Hodgkin lymphoma (27%).

## Discussion

It is a challenging yet opportune time for practitioners delivering end-of-life care, especially in the UK where there is a drive to enable patients to be cared for and die in the place of their choice. Our review clearly illustrates that the majority of patients with a haematological malignancy die in hospital. They are in fact more than twice as likely to die in hospital as those with other cancers. Studies mostly originated from within Europe, the USA and Australia and findings were generally consistent across these areas. Given that home is usually considered to be the preferred place of death [7,8], this has implications for both patients, who may not be dying in their preferred place, and also health commissioners, who may be funding costly end-of-life care in inappropriate, acute hospital settings.

In order to ensure haematology patients receive appropriate end-of-life care, and are enabled to die in their preferred place, it is crucial that an evidence base is established so that existing practices can be defined and evaluated. In terms of understanding why hospital deaths are so common, current explanations are often derived from anecdote, case studies or qualitative research studies. Factors cited as leading to hospital death often include the complex transition from an active or curative approach to a palliative approach to care - a transition which is not always clear in haematology [6,35-38]. This lack of clarity can arise because some haematological diseases, such as myeloma and follicular lymphoma are considered incurable from diagnosis; thus all treatments, although having the potential to substantially prolong life, are essentially given with palliative intent from the outset. The situation is further complicated by the increasing number of salvage therapies available to patients, resulting in continued treatment even in the very late stages of disease, a situation which may give rise to sentiments of denial or the continued hope of a response, both in patients and practitioners [13,36,39].

In the UK, initiatives such as Advanced Care Planning, Preferred Priorities for Care and the Gold Standards Framework [11] aim to provide patients with the opportunity to discuss the place of their death and plan where they would prefer this to happen. However, the patient pathway associated with haematological malignancy is recognised as being complex and uncertain, with death sometimes occurring suddenly, unexpectedly, or very rapidly after diagnosis [26,39-41]. Also, certain treatments, such as allogeneic bone marrow transplantations, can be associated with a rapid change in the focus of care from curative to palliative [42]. In contexts such as these the opportunity to discuss place of death may not arise, or may even be inappropriate. Such situations may lead to difficulties estimating prognosis and concerns about the inappropriate early withdrawal of treatment [38]. Failure to recognise and respond to this transition may, however, lead to a crisis management situation and an inappropriate emergency hospital admission [41].

Further factors influencing place of death are the disease symptoms and the side effects of chemotherapy, which our previous work on patient pathways has shown can be intensive and prolonged [5]. The possibility of complicating factors, including anaemia, bleeding and infection, can result in the need for ongoing transfusion of blood products, antibiotic and antifungal medication, which may require long term hospitalisation, with death in acute or intensive care settings often being reported [13,32,35,36,43].

In some situations, an excess of hospital deaths among haematological malignancy patients has been suggested to reflect the fact that haematologists have limited links with palliative care services. This lack of integration has been recognised as a particular problem in Australia, [6,38,39,44,45] and has led to the UK [46] making recommendations to promote links between these specialities. However, because of the nature of their condition, haematology patients are known to have a sustained and intense relationship with their haematology team [22,32,41]. Indeed, unlike patients with other types of cancer, those in haematology are often managed throughout the entire course of their illness, including the terminal phase, within haematology [30]. Whilst this close association may explain the apparent lack of palliative care involvement it could also result in patients having difficulty accessing community palliative care services or hospice facilities - a situation which may lead to an inappropriate acute hospital admission for terminal care and ultimately death [41].

Our review indicated a dearth of empirical research exploring these issues, in particular the factors and circumstances either leading to emergency hospital admission at the end-of-life, or preventing hospital discharge and the delivery of end-of-life care at home. This may for example include a better understanding of the degree to which dependence on blood product transfusions exists and potential alternatives to receiving these products in hospital [41]. Clearly, further work exploring the complexity and duration of the patient pathway, variations in the type and intensity of treatment and the transition from active, life prolonging to palliative care would lead to better understanding of end-of-life issues among haematology patients.

End-of-life care is a complex, multi-disciplinary and indeed multi-dimensional concept, with many interrelated themes that cannot be considered in isolation [47]. It includes the provision of care by multiple practitioners (for example, clinicians and nurse specialists in haematology, palliative care and other medical specialities); liaison and interaction between different settings (primary and secondary care, out of hours care, social care, care homes and hospices) and services (NHS, voluntary and local authority). Thus a greater understanding is also needed about the broader organisation, management and delivery of end-of-life care for haematology patients. More information is also needed about potential limiting factors, such as a lack of community facilities and resources including hospice places, community staff, equipment or training. Importantly, variations in place of death by haematological subtype [30] emphasise the need to further explore these differences by specific disease classifications.

Although existing research indicates that preferred place of death is often home, it is important to recognise that currently there is no empirical evidence to support this in haematology and again this is an area where more research is needed. If, as the literature suggests, patients develop a strong relationship with their clinical haematology team over the duration of their illness, and hospital is regarded as a place where they feel secure and are most likely to receive prompt medical attention, then hospital may indeed be the preferred place of place of care and death for some. Further research with patients and carers is needed to develop detailed, clearer insight into these issues.

## Conclusion

Home is generally considered the preferred place of death but haematology patients usually die in hospital. This has implications for patients who may not be dying where they wish, and also health commissioners who may be funding costly end-of-life care in inappropriate acute hospital settings. More research is needed about preferred place of care for haematology patients, reasons for hospital deaths, and how these can be avoided if home death is preferred.

## Competing interests

The authors declare that they have no competing interests.

## Authors' contributions

The Palliative Care and Haematological Malignancy Steering Committee had the original idea for the review. DH managed the review, conducted the search, retrieved, scored and performed the final screening of studies, extracted data and wrote the manuscript. HC independently second-scored the studies. ER and AS performed the final screening of studies, extracted data and calculated risk estimates. All authors commented on the final version of the manuscript.

## Pre-publication history

The pre-publication history for this paper can be accessed here:

http://www.biomedcentral.com/1472-684X/9/9/prepub
